# HER-2 Expression in Colorectal Cancer and Its Correlation with Immune Cell Infiltration

**DOI:** 10.3390/biomedicines11112889

**Published:** 2023-10-25

**Authors:** Di Yang, Bo Wang, Yinuo Li, Jingyao Zhang, Xuantong Gong, Hao Qin, Yan Wang, Yahui Zhao, Yong Wang

**Affiliations:** 1 Department of Ultrasound, National Cancer Center, National Clinical Research Center for Cancer, Cancer Hospital, Chinese Academy of Medical Sciences and Peking Union Medical College, Beijing 100021, China; yangdi4134@163.com (D.Y.); dorwangbo@163.com (B.W.); gongxuantong@163.com (X.G.); 2 State Key Laboratory of Molecular Oncology, National Cancer Center, National Clinical Research Center for Cancer, Cancer Hospital, Chinese Academy of Medical Sciences and Peking Union Medical College, Beijing 100021, China; liyinuo1027@126.com; 3 Key Laboratory of Cancer and Microbiome, State Key Laboratory of Molecular Oncology, National Cancer Center, National Clinical Research Center for Cancer, Cancer Hospital, Chinese Academy of Medical Sciences and Peking Union Medical College, Beijing 100021, China; bessiezhang05@163.com (J.Z.); pkuqinhao@126.com (H.Q.); yanwang@cicams.ac.cn (Y.W.)

**Keywords:** HER2, colorectal cancer, immune cell

## Abstract

Background: This study aimed to investigate the effect of increased HER-2 expression on tumor-infiltrating lymphocytes (TILs) and determine its impact on the prognosis of colorectal cancer (CRC) patients; Methods: HER-2, CD4, CD8, CD19, LY6G, CD56, CD68, CD11b, and EpCam expression in CRC tissues and adjacent paracancerous tissues were assessed using multiplex fluorescence immunohistochemical staining. The correlation between HER-2 expression and the number of TILs in CRC tissues was analyzed. Kaplan–Meier and Cox proportional hazards models were used to analyze survival outcomes; Results: The expression of HER-2 in tumor tissues was higher than that in paracancerous tissues (1.31 ± 0.45 vs. 0.86 ± 0.20, *p* < 0.05). Additionally, there was an increase in the numbers of CD4+, CD8+, CD19+, and CD68+ cells in CRC tissues (14.11 ± 1.10 vs. 3.40 ± 0.18, *p* < 0.005; 0.16 ± 0.12 vs. 0.04 ± 0.04, *p* < 0.005; 0.71 ± 0.46 vs. 0.25 ± 0.13, *p* < 0.0005; 0.27 ± 0.24 vs. 0.03 ± 0.11, *p* < 0.05). An increase in HER-2 expression was positively correlated with an increase in CD4, CD8, and CD19 (*p* < 0.0001). In HER-2-positive CRC tissues, CD68 expression was increased (0.80 ± 0.55 vs. 0.25 ± 0.22, *p* < 0.05). In HER-2-upregulated CRC tissues, CD4, CD8, CD19, CD68, CD11b, Ly6G, and CD56 expressions were elevated (0.70 ± 0.37 vs. 0.32 ± 0.17, *p* = 0.03; 0.22 ± 0.13 vs. 0.09 ± 0.06, *p* = 0.03; 0.31 ± 0.19 vs. 0.12 ± 0.08, *p* = 0.02; 1.05 ± 0.62 vs. 0.43 ± 0.21, *p* < 0.01; 1.34 ± 0.81 vs. 0.53 ± 0.23, *p* < 0.01; 0.50 ± 0.31 vs. 0.19 ± 0.10, *p* < 0.01; 1.26 ± 0.74 vs. 0.52 ± 0.24, *p* < 0.01). Furthermore, increased HER-2 expression was an independent risk factor for recurrence-free survival (RFS) in patients (*p* < 0.01, HR = 3.421); Conclusions: The increased expression of HER-2 and its relationship with immune cells will provide new insights for immunotherapy in CRC patients.

## 1. Introduction

Colorectal cancer (CRC) ranks as the third most common cancer and the second leading cause of cancer-related deaths worldwide [1]. According to the most recent data on population-based cancer occurrence and outcomes compiled by the American Cancer Society, the mortality of CRC is increasing among young adults: from 2005 to 2019, the death rate rose by 1.2% per year in individuals younger than 50 years and by 0.6% per year in those aged 50 to 54 years [2].

Human epidermal growth factor receptor 2 (HER-2) is a transmembrane protein with tyrosine-protein kinase activity, playing a crucial role in promoting biological processes such as cell proliferation, growth, and differentiation. Reports show a variability in the proportion of HER-2 positivity in CRC, with membrane expression ranging from 2% to 11% [3,4]. HER-2 positivity is associated with tumor development and an increased risk of metastasis, making it a routine biomarker and significant therapeutic target in cancer treatment. Following successful anti-HER-2 therapies for breast and gastric cancers [5,6,7], researchers have begun exploring its potential in CRC treatment. For example, a proof-of-concept, multicenter, open-label, phase 2 trial done at four Italian academic cancer centers evaluated the efficacy of dual HER-2 blockade with trastuzumab and lapatinib in inhibiting tumor growth in patient-derived xenografts of HER2-amplified metastatic colorectal cancer. This approach proved to be active and well-tolerated [8]. In 2021, trastuzumab-emtansine (T-DXd) was tested in a phase 2 trial targeting HER-2 expressing metastatic CRC, with 23 out of 53 enrolled patients showing objective responses (objective response rate: 45.3%) and acceptable side effects [9]. However, as of now, no therapies have been approved for use in this patient population [10].

It is noteworthy that various HER-2-targeted therapies have been effective in treating HER2 overexpressing breast, gastric, and esophageal cancers, and they also appear to exert their effects through immunological mechanisms or favorable anti-tumor immunomodulation [11]. Meanwhile, growing evidence suggests that immune infiltration can better define patient responses to immunotherapy [12,13]. Among different subtypes, HER-2-enriched breast cancers are the most immunogenic tumors [14,15]. They exhibit the highest levels of tumor-infiltrating lymphocytes (TILs), which are associated with high expression of immune-activating genes [16]. However, our knowledge about TILs in the context of HER-2 expression in CRC is limited. Furthermore, HER-2 overexpression is associated with poor prognosis, high mortality, and increased recurrence and metastasis in other cancers, but its prognostic value in CRC remains unclear [17]. Hence, this study was conducted to analyze the characteristics of immune cells in the TILs of HER-2-positive and upregulated CRC patients. By exploring the immune microenvironment and clinicopathological characteristics of these individuals, this research aims to provide new directions for their treatment.

## 2. Materials and Methods

### 2.1. Clinical Samples

Tumor and paracancerous tissues were collected from 100 consecutive patients treated at the National Cancer Center, Cancer Hospital, and Chinese Academy of Medical Science between 1 October 2017 and 31 March 2019. Inclusion criteria: all patients were diagnosed with adenocarcinoma and underwent radical resections at the Cancer Hospital, Chinese Academy of Medical Sciences; no preoperative anti-tumor therapy such as radiotherapy, chemotherapy, or immunotherapy; patients were older than 18 years; all patients provided written informed consent. Exclusion criteria: incomplete patient clinical and pathologic information. Consequently, 97 patients were evaluated. A flowchart of patient enrollment and grouping is shown in Figure S1 of the Supplementary Material. All recruited patients were consistently followed up after tumor resection to determine recurrence-free survival (RFS), calculated from the curative resection date to the tumor recurrence diagnosis. Preoperative and tumor recurrence diagnoses adhered to the guidelines outlined in the Chinese protocol of diagnosis and treatment of colorectal cancer of the National Health Commission [18]. This study was approved by the ethics committee of the Cancer Hospital, Chinese Academy of Medical Sciences (NCC-001723) [19].

### 2.2. Immunohistochemistry (IHC)

HER-2 expression results were obtained by reviewing postoperative pathological immunohistochemistry, and were corrected again by 2 pathologists according to the HERACLES diagnostic criteria [20]: IHC 3+: intense circumferential, basolateral, or lateral staining in ≥50% of cells; IHC 2+: moderate circumferential, basolateral, or lateral staining in ≥50% of cells; IHC 1+: faint segmental or granular staining with any cellularity; IHC 0: no staining. HER-2 positivity was indicated by IHC 3+, while IHC 1+ and IHC 0 were considered negative. For individuals with IHC 2+, further in situ hybridization (ISH) was performed. A HER-2/CEN17 ratio ≥2 in ISH was considered HER-2-positive. Then, we screened out those with higher expression of HER-2 in CRC tissue compared with the corresponding paracancerous tissue to obtain the upregulation multiple of HER-2. To show the significance of HER-2 upregulation, we set the upregulation cutoff value to 1.5. Then, the HER-2-upregulated and non-upregulated CRC tissues were divided into two groups.

### 2.3. Immunofluorescence Staining

Tumor tissues were fixed in 4% paraformaldehyde and immuno-fluorescence was performed on 5 μm thick paraffin sections after heat-induced antigen retrieval. The following primary antibodies were used: CD4 (1:200, Abcam, Cambridge, UK, ab34276), CD8 (1:200, Abcam, ab101500), CD19 (1:200, CST, #90176s), CD56 (1:200, Abcam, ab21336), CD68 (1:200, Abcam, ab53444), CD11b (1:200, Abcam, ab133357), LY6G (1:200, Abcam, ab25377), and EpCam (1:200, Abcam, ab213500). The HER-2 immunohistochemical reagent was the ready-to-use 4B5 rabbit anti-human monoclonal antibody, purchased from Roche. Subsequently, we incubated the slides with fluorescent secondary antibodies (fluorescein (FITC)-conjugated goat anti-rabbit IgG (H+L), 1:1000, Proteintech, San Diego, CA, USA, SA00003-2; rhodamine (TRITC)-conjugated goat anti-rat IgG (H+L), 1:1000, Proteintech, SA00007-7; Alexa Fluor^®^ 647-conjugated goat anti-mouse IgG (H+L), 1:1000, Cell Signaling Technology, Danvers, MA, USA, #4410), DAPI (Thermo Scientific, Waltham, MA, USA, #62247), and the Opal 6-Color Manual IHC Kit (Akoya Biosciences, Marlborough, MA, USA, NEL811001KT) for multilabel immunofluorescence analysis. Positive cells were determined and quantified by Perkin Elmer (Waltham, MA, USA) in FormTM system.

### 2.4. Quantification and Statistical Analysis

GraphPad Prism 8.0 was used for all statistical analyses. Unpaired Student’s *t*-test and ANOVA were used for comparison of continuous variables between two and multiple groups. Comparison of rank data was performed using a non-parametric rank sum test of two independent samples. Correlation analysis was performed using Spearman’s correlation coefficient. Univariate and multivariate Cox proportional hazards regression identified independent prognostic factors for tumor recurrence. Quantitative data are shown as mean ± S.D. A difference of *p* < 0.05 is statistically significant.

## 3. Results

### 3.1. Increased HER-2 Expression and Increased Number of Immune Cells in CRC Tissues Compared to Paracancerous Tissues

**Figure 1:**
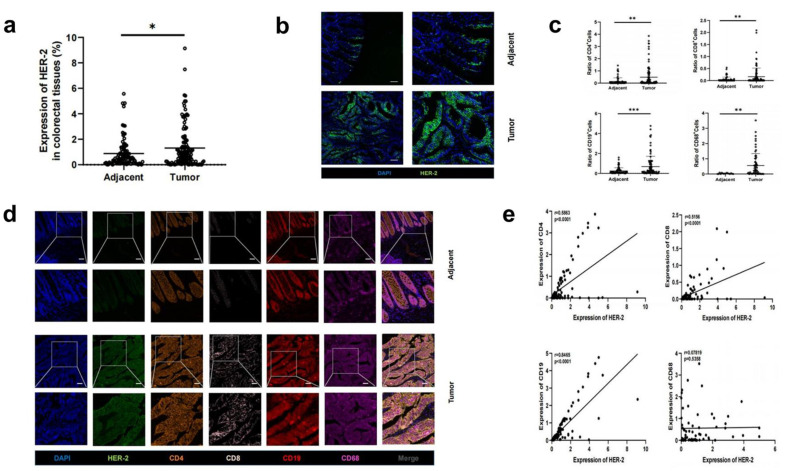
HER-2 expression and HER-2 co-localization with immune cells in CRC tissue and paracancerous tissue. (**a**) Increased expression of HER-2 in CRC tissues. (**b**) HER-2 immunofluorescence in CRC tissue and paracancerous tissue. Scale bar: 100 μm. (**c**) Increased CD4, CD8, CD19, and CD68 expression in CRC tissues. (**d**) CD4, CD8, CD19, and CD68 co-localize with HER-2 immunofluorescence in cancerous versus paraneoplastic tissues. Scale bar: 100 μm. (**e**) HER-2 expression showed positive correlations with CD4, CD8, and CD19, respectively. *: *p* < 0.05, **: *p* < 0.005, ***: *p* < 0.0005.

First, the expression of HER-2 in CRC tissues and paracancerous tissues was examined. Samples from CRC tissues had significantly more HER-2 expression than those from paracancerous tissues (1.31 ± 0.45 vs. 0.86 ± 0. 20, *p* < 0.05) (Figure 1a,b). Next, we included multilabel immunofluorescence (MIF) of HER-2, CD4, CD68, CD19, LY6G, CD56, CD68, CD11b, and EpCam in CRC tissue array. Cancerous lesions had more infiltration of CD4+ cells and CD8+ cells compared to normal tissue (14.11 ± 1.10 vs. 3.40 ± 0.18, *p* < 0.005; 0.16 ± 0.12 vs. 0.04 ± 0.04, *p* < 0.005); CD68+ cells and CD19+ cells displayed a similar pattern (0.27 ± 0.24 vs. 0.03 ± 0.11, *p* < 0.05; 0.71 ± 0.46 vs. 0.25 ± 0.13, *p* < 0.0005) (Figure 1c,d); and CD4, CD8, and CD19 were found to be elevated, with increasing HER-2 expression by correlation analysis (Figure 1e).

### 3.2. Increased HER-2 Expression in CRC Tissues Is Accompanied by Increased Infiltration of Immune Cells

**Figure 2:**
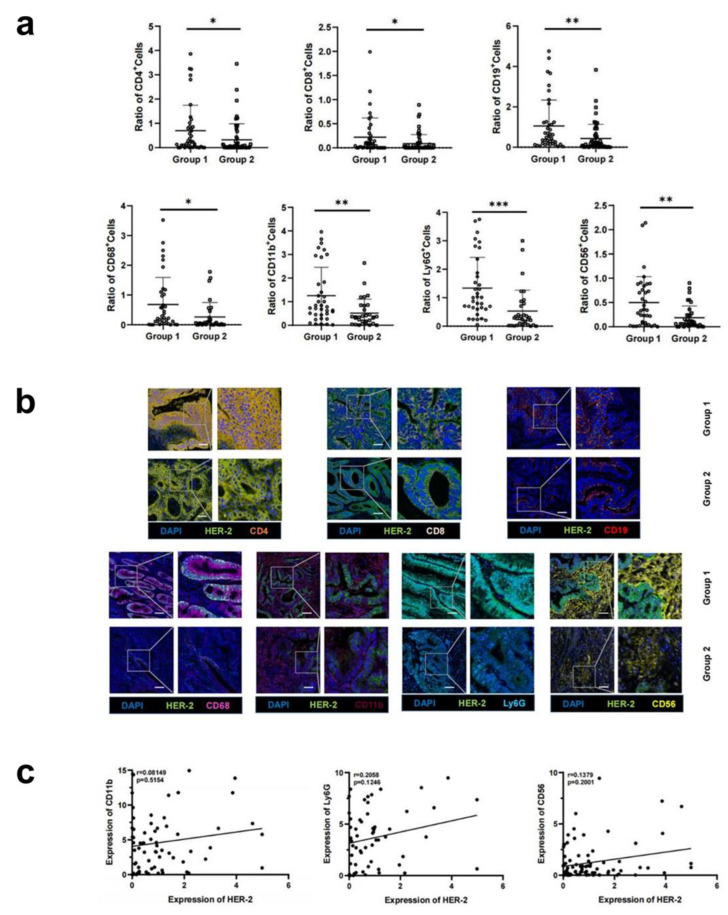
Relationship between increased HER-2 expression and immune cell infiltration in CRC tissue. (**a**) Expression of CD4, CD8, CD19, CD68, CD11b, Ly6G, and CD56 was higher in the HER-2-upregulated group (group 1) compared to the non-upregulated group (group 2). (**b**) Immunofluorescence showed an increase in the number of CD4, CD8, CD19, CD68, CD11b, Ly6G, and CD56+ cell infiltrates in group 1 compared to group 2. Scale bar: 100 μm. (**c**) Correlation analysis between CD11b, Ly6G, and CD56 and increased HER-2 expression. *: *p* < 0.05, **: *p* < 0.005, ***: *p* < 0.0005.

We divided CRC tissues into HER-2-negative and positive groups, then counted the differences in the expression of the above immune cell markers in the two groups separately. After determining HER-2 IHC 2+ tissue by ISH, they were all found to be HER-2-negative. Therefore, a total of three cases in this study sample ended up with positive HER-2 expression and the rest with negative expression. The result showed that the proportion of CD68+ cells was higher in HER-2-positive CRC tissues (0.80 ± 0.55 vs. 0.25 ± 0.22, *p* < 0.05) (Supplementary Figure S2a,b). Additionally, the upregulation of HER-2 was calculated, and the infiltration of immune cells was compared separately in HER-2-upregulated and non-upregulated groups. It turned out that HER-2 expression was upregulated in 43.3% of CRC tissues, with an average upregulation fold of 13.85, whilst a significant increase in the infiltration of CD4+, CD8+, CD68+, CD19+, LY6G+, CD56+, and CD11b+ cells occurred in the HER-2-upregulated group (Table 1) (Figure 2a,b). Correlation analysis showed no correlation between CD11b, Ly6G, and CD56 and increased HER-2 expression (Figure 2c).

### 3.3. Upregulation of HER-2 Expression Is a Risk Factor for Prognosis in CRC Patients

**Figure 3:**
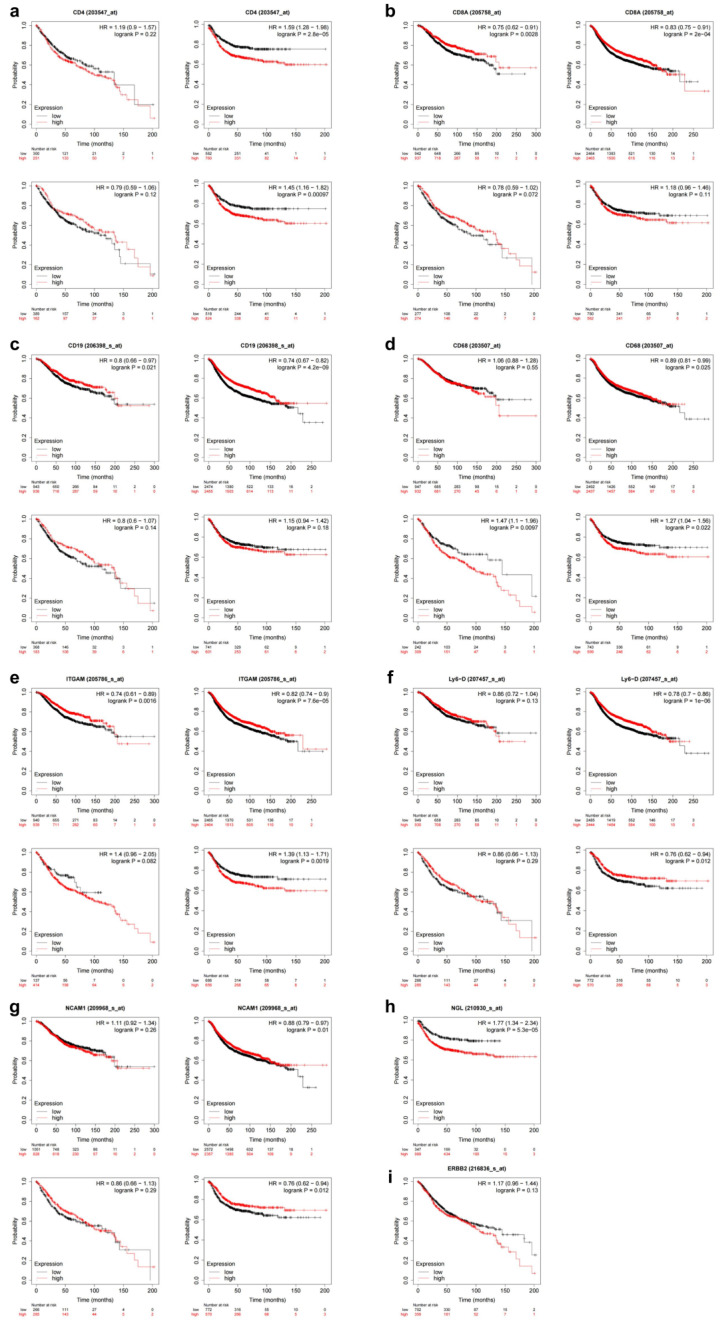
Kaplan–Meier survival curves for CRC patients with or without high expression levels of CD4, CD8, CD19, CD68, CD11b, Ly6G, CD56, and HER-2 in the Kaplan–Meier plot (https://kmplot.com/analysis/index.php?p=service&cancer=colon, accessed on 9 June 2023). (**a**–**g**): Effect of CD4, CD8, CD19, CD68, CD11b, Ly6G and CD56 co-expression with HER-2 on OS and RFS, respectively. (**h**): HER-2 Expression and Its Impact on RFS. (**i**): HER-2 Expression and Its Impact on OS.

We summarized the basic clinical characteristics of 97 CRC patients (Table 2, Supplementary Table S1). The median follow-up time for patients in this study was 54.5 months. There were 90 recurrence-free survivors at 1 year, with an RFS rate of 92.8%; 85 recurrence-free survivors at 3 years, with an RFS rate of 87.6%; and 73 recurrence-free survivors at 5 years, with an RFS rate of 75.3%. After 5 years of follow-up, a total of 24 people in this study experienced tumor recurrence and a total of 10 people died. Notably, only three of the included cases were HER-2-positive and all were alive at follow-up, resulting in no statistical difference in survival curves between HER-2-positive and negative patients. We incorporated the age, gender, tumor position, TNM stage, lymphovascular invasion, large vessel invasion, tumor budding, peripheral nerve invasion, CEA, HER-2, tumor differentiation, and chemotherapy into univariate Cox regression for RFS. The results revealed that TNM stage (*p* = 0.037, *p* = 0.001, *p* = 0.001 respectively), CEA (*p* = 0.004), and HER-2 (*p* = 0.047) were influential factors in RFS. Increased TNM stage, elevated CEA levels, and HER-2 upregulation were associated with a higher risk of tumor recurrence. Furthermore, multivariate Cox regression analysis indicated that N stage (hazard ratio [HR] = 3.238, 95% confidence interval [CI]: 1.648–6.363, *p* = 0.001), M stage (HR = 97.428, 95% CI: 5.881–1613.942, *p* = 0.001), and HER-2 (HR = 3.421, 95% CI: 1.359–8.613, *p* = 0.009) were independent prognostic factors for RFS (Table 3). Survival analysis was performed for both overall survival (OS) and RFS using the Kaplan–Meier plotter [21]. The results for the best performing cutoff were exported for each gene in a separate database, and these were used to generate Kaplan–Meier plots to visualize the correlation between gene expression and survival [22]. The results of the left side of each group were observed with OS as the observation endpoint and of the right side with RFS as the observation endpoint to observe the prognostic impact of the expression of immune cell markers and the changes in prognosis produced by immune cells co-expressing with HER-2 in patients. The results showed that high CD4 expression adversely affected RFS (logrank *p* = 2.8 × 10^−5^ (Figure 3a); CD68, Ly6G, and CD56 were protective against RFS (logrank *p* = 0.03; logrank *p* = 1.0 × 10^−6^; logrank *p* = 0.01) (Figure 3d,f,g); and high expression of CD8, CD19, and CD11b were protective factors for both OS and RFS of patients (OS: logrank *p* = 0.003, RFS: logrank *p* = 2.0 × 10^−4^; OS: logrank *p* = 0.02, RFS: logrank *p* = 4.2 × 10^−9^; OS: logrank *p* = 0.002, RFS: logrank *p* = 7.6 × 10^−5^;) (Figure 3b,c,e). HER-2 expression was also included as an influencing factor, and it was found that both high CD4 and HER-2 expression and high CD11b and HER-2 expression were detrimental to patients’ RFS (logrank *p* = 0.001; logrank *p* = 0.002) (Figure 3a,e), and high CD68 and HER-2 expression were risk factors for both OS and RFS (OS: logrank *p* = 0.01, RFS: logrank *p* = 0.02) (Figure 3d), while high Ly6G and HER-2 expression and high CD56 and HER-2 expression were protective for RFS (logrank *p* = 0.012; logrank *p* = 0.012) (Figure 3f,g). Finally, the effects of HER-2 expression on patients’ OS and RFS were analyzed. High HER-2 expression was found to be an adverse factor for RFS (log-rank *p* = 5.3 × 10^−5^), while it had no significant effect on OS in this experimental group (*p* = 0.13) (Figure 3h,i).

## 4. Discussion

Currently, the incidence and mortality rates of colorectal cancer are among the highest in the world, imposing a substantial burden on healthcare systems. Traditional treatments such as surgery, radiotherapy, and chemotherapy have limitations in addressing the challenges faced by colorectal cancer patients, including a high incidence of relapse and severe side effects. Therefore, there is an urgent need to develop new treatment strategies.

HER-2, a member of the human epidermal growth factor receptor family, is expressed in various malignancies. The 2021 edition of the National Comprehensive Cancer Network (NCCN) Clinical Practice Guidelines for Colorectal Cancer recommends HER-2 testing for CRC and the use of anti-HER-2 therapies. These therapies, including trastuzumab in combination with patuximab or lapatinib, are recommended for RAS and BRAF wild-type HER-2 amplified CRC. Anti-HER-2 treatment is emerging as a promising therapeutic target for CRC [23,24]. Previous reports have indicated that HER-2 has malignant transforming activity, and high expression of HER-2 can serve as a reliable indicator of colorectal cancer prognosis. It often suggests a poorer prognosis and shorter survival for CRC patients [25,26]. The 5-year survival rate of HER-2-positive patients is significantly lower than that of patients with negative HER-2 expression [27]. However, a meta-analysis conducted by Li et al. [28] found that HER2 overexpression may have a limited impact on the survival of colorectal cancer patients. Interestingly, we also observed that increased HER-2 expression did not lead to reduced overall survival time but was associated with a higher recurrence rate. This discrepancy might be due to increased HER-2 expression resulting in tumors with a faster growth rate and higher aggressiveness. Additionally, most patients in our study underwent postoperative chemotherapy, which may have primarily been focused on controlling the growth of recurrent tumors.

Furthermore, the malignant phenotype of a tumor is determined not only by the intrinsic activity of the tumor but also by the immune cells activated by recruitment in the TME. In HER-2-positive breast cancer, the TME has emerged as both a potential prognostic factor and a modulator of treatment response. Different tumor subtypes have different immunogenicity [29,30]. HER-2-positive breast cancer is generally considered to be more immunogenic than hormone receptor-positive and HER-2-negative breast cancer, although it is less immunogenic than triple-negative breast cancer. Moreover, certain chemotherapeutic agents like anthracyclines and cyclophosphamide, along with HER-2-targeted therapies, can activate the immune system through processes such as immunogenic cell death and antibody-dependent cytotoxicity [31,32]. Additionally, concurrent administration of paclitaxel and trastuzumab can enhance the immune effects of trastuzumab by acting on tumor cells and natural killer cells [33]. These findings highlight the critical role of TME in modulating the immune response and therapeutic efficacy in HER-2-positive breast cancer.

Similar associations are emerging in colorectal cancer (CRC) research [34]. The composition and function of tumor-infiltrating lymphocytes (TILs) in CRC are altered in response to the host’s immune status. These alterations can make TILs more susceptible to drug interventions. For instance, trastuzumab has been shown to enhance the efficacy of immunotherapy by inducing TILs [35]. Research into these mechanisms has revealed complex interactions between TILs and tumor cells. Prior studies have shown that the immune system can play a dual role in supporting defense and promoting tumor progression. For example, Garaud et al. [36] discovered an increase in tumor-infiltrating B cell density in untreated HER-2-positive primary breast cancer compared with normal breast tissues, which is associated with higher tumor grades, higher proliferation and hormone receptor negativity. Moreover, in CRC, the mismatch repair (MMR) system plays a significant role. MMR ensures high-fidelity DNA replication, maintains genomic stability, and reduces spontaneous mutations [37]. Fan et al. [38] concluded that in CRC patients with deficient MMR (dMMR) or microsatellite instability-high (MSI-H) tumors, approximately 15% of cases exhibit major histocompatibility complex (MHC) class I peptide complexes on the tumor cell surface. These complexes include mutated peptides recognized as neoantigens, which promote the priming and infiltration of immune cells. Specifically, CD8+TILs, CD4+TILs, and macrophages migrate into the TME, eliciting interferon-gamma (IFNγ) secretion and anti-tumor effects [39]. High levels of tumor infiltration by activated CD8+TILs correlate with better survival among CRC patients. However, macrophage infiltration is associated with a poor prognosis in CRC. The high invasiveness of tumor-associated macrophages (TAMs) is linked to reduced overall survival in several types of cancer [40]. In a retrospective study of 123 patients with advanced colorectal cancer, patients with low CD68+ TAMs showed significantly higher rates of recurrence and shorter OS [41]. Moreover, some studies have revealed that macrophages in TME predominantly exhibit an M2 phenotype, which promotes tumor development and can facilitate colorectal cancer metastasis [42,43]. The increase in macrophages in HER-2-positive CRC tissues with HER-2-upregulated CRC tissues in this study serves as a clear indicator of heightened danger signals.

In gastric cancer, Yuan et al. [44] emphasized the pivotal role of HER-2 in the immune microenvironment through pan-cancer analysis. Their findings indicated that HER-2 expression was inversely correlated with the infiltration levels of various immune cells, such as M1 macrophages, M2 macrophages, myeloid dendritic cells, cancer-associated fibroblasts, naïve CD4+ T cells, CD8+ T cells, gdT cells, T helper 2 cells, and B cells, but positively correlated with the infiltration levels of M0 macrophages, neutrophils, memory CD4+ T cells, and T follicular helper cells. Our study sheds light on novel TIL infiltration patterns following increased HER-2 expression in CRC. Here, tumor cells express HER-2 protein as an antigen that can be recognized and attacked by the immune system, enhancing the immunogenicity of the tumor. Simultaneously, HER-2 triggers the proliferation and aggressiveness of tumor cells, leading to the release of tumor-associated antigens. This indirectly activates the immune system, resulting in heightened TILs. This is corroborated by our results, which demonstrate an increase in the number of infiltrating CD4+ T cells, CD8+ T cells, and B cells with higher HER-2 expression. However, it is important to note that while immune cells exhibit greater infiltration in HER-2-positive CRC tissues, this does not necessarily translate to improved patient prognosis. Immune cells and factors within the TME can also exert immunosuppressive and tumor-promoting effects. For instance, a breast cancer-related study found that TAMs are the main source of IL-8 in TME and that HER-2-positive patients have significantly higher serum levels of IL-8 compared to HER-2-negative breast cancer patients [45]. Furthermore, it has been demonstrated that TAMs isolated from TME with invasive phenotypic inflammatory breast carcinomatosis are characterized by overexpression and secretion of IL-8, which induces the movement and invasion of breast carcinomatosis cell lines [46].

The expression of HER-2 positivity in the cases collected for this study was consistent with previous reports. Although the rate of HER-2 positivity in CRC patients was lower than that in breast and gastric cancers, our data, in conjunction with information from public databases, clearly demonstrated that increased HER-2 expression was significantly linked to a higher rate of recurrence in CRC. This finding establishes HER-2 expression as an independent risk factor for tumor recurrence in CRC patients. Meanwhile, our study also revealed that increased HER-2 expression was closely associated with enhanced infiltration of immune cells within the TME. This heightened immune cell presence contributed to an increased immunogenicity of the tumor. In essence, CRC, traditionally considered a “cold tumor” with limited immune cell infiltration, seemed to transition into a “hot tumor” with increased HER-2 expression. This observation supports the need for further clinical evaluation of immunotherapy approaches in the treatment of such tumors. However, it is important to acknowledge the limitations of our study, including a relatively small sample size and a limited number of HER-2-positive patients. These factors resulted in the absence of OS outcomes in the comparison sample. In addition, IHC, as a semi-quantitative method to determine HER-2 expression, may lead to some false negatives or false positives.

In conclusion, this study uncovered a novel characteristic of immune cell behavior in the context of increased HER-2 expression in CRC. Traditionally, high expression of HER-2 implies a poor prognosis for CRC patients, whereas our findings suggest that increased HER-2 expression significantly affects RFS. Notably, we observed a positive correlation between HER-2 expression and increased infiltration of T cells and B cells in the TME. In addition, the number of T cells, B cells, macrophages, and intrinsic immune cells increased in CRC tissues with upregulated HER-2 expression. These findings open up promising possibilities for the treatment of this patient population, and our future research will delve into the potential use of these immune cells for therapeutic purposes.

## Figures and Tables

**Table 1:** Immune cell infiltration in HER-2-upregulated and non-upregulated groups.

Cell	Mean ± SD		*t*-Test	
	HER-2-Upregulated	HER-2-Non-Upregulated	*t*	*p*
CD4+	0.70 ± 0.37	0.32 ± 0.17	2.15	0.03
CD8+	0.22 ± 0.13	0.09 ± 0.06	2.18	0.03
CD68+	0.31 ± 0.19	0.12 ± 0.08	2.41	0.02
CD19+	1.05 ± 0.62	0.43 ± 0.21	3.02	<0.01
LY6G+	1.34 ± 0.81	0.53 ± 0.23	3.58	<0.01
CD56+	0.50 ± 0.31	0.19 ± 0.1	3.20	<0.01
CD11b+	1.26 ± 0.74	0.52 ± 0.24	3.11	<0.01

**Table 2:** Summary of colorectal cancer patients’ demographic and clinical characteristics (*n* = 97).

Factor	Variables	Proportion
Age	≥60	50 (51.5%)
	<60	47 (48.5%)
Gender	Male	57 (58.9%)
	Female	40 (41.2%)
Tumor position	Ascending colon	12 (12.4%)
	Transverse colon	1 (1.0%)
	Descending colon	11 (11.3%)
	Sigmoid colon	25 (25.8%)
	Rectum	48 (49.5%)
T Stage	1	3 (3.1%)
	2	21 (21.6%)
	3	42 (43.3%)
	4	31 (32.0%)
N Stage	0	49 (50.5%)
	1	30 (30.9%)
	2	18 (18.6%)
M Stage	0	94 (96.9%)
	1	3 (3.1%)
Lymphovascular invasion	Yes	32 (33.0%)
	No	65 (67.0%)
Large vessel invasion	Yes	15 (15.5%)
	No	82 (84.5%)
Tumor budding	Yes	24 (24.7%)
	No	73 (75.3%)
Peripheral nerve invasion	Yes	32 (33.0%)
	No	65 (67.0%)
CEA (0~5 ng/mL)	<5	59 (60.8%)
	≥5	38 (39.2%)
HER-2	−	48 (49.5%)
	+	39 (40.2%)
	++	7 (7.2%)
	+++	3 (3.1%)
Differentiation	1	9 (9.3%)
	2	20 (20.6%)
	3	64 (66.0%)
	4	2 (2.1%)
	5	2 (2.1%)
Chemotherapy	Yes	83 (85.6%)
	No	14 (14.4%)
5-year recurrence-free survival (Recurrence event = 1)	0	73 (75.3%)
	1	24 (24.7%)

**Table 3:** Variate Cox analyses for RFS of HER-2-upregulated group and non-upregulated group.

Variables	Univariate Analysis		Multivariate Analysis	
	*p*	HR (95% CI)	*p*	HR (95% CI)
Age	0.485	0.987 (0.953–1.023)		
Gender	0.651	1.208 (0.533–2.736)		
Tumor position	0.583	1.093 (0.794–1.506)		
T stage	0.037	1.804 (1.024–3.179)	0.924	1.031 (0.554–1.919)
N stage	0.001	2.454 (1.434–4.199)	0.001	3.238 (1.648–6.363)
M stage	0.001	13.182 (2.777–62.578)	0.001	97.428 (5.881–1613.942)
Lymphovascular invasion	0.918	1.048 (0.433–2.534)		
Large vessel invasion	0.102	2.269 (0.827–6.224)		
Tumor budding	0.100	2.185 (0.861–5.547)		
Peripheral nerve invasion	0.078	2.104 (0.921–4.806)		
CEA (0~5 ng/mL)	0.004	1.007 (1.002–1.011)	0.838	0.999 (0.992–1.007)
HER-2	0.047	2.317 (1.011–5.309)	0.009	3.421 (1.359–8.613)
Differentiation	0.426	0.807 (0.476–1.368)		
Chemotherapy	0.513	0.698 (0.238–2.048)		

## Data Availability

All data produced and analyzed for this study are available from the corresponding author upon reasonable request.

## Supplementary Materials

The following supporting information can be downloaded at: https://www.mdpi.com/article/10.3390/biomedicines11112889/s1, Figure S1: Patient intake and grouping flowchart; Figure S2: Expression of CD68 in HER-2-positive and HER-2-negative CRC tissues; Table S1: Clinical features of CRC patients.
