# Supplementary material for: HER-2 Expression in Colorectal Cancer and Its Correlation with Immune Cell Infiltration
Source: Biomedicines. 2023 Oct 25;11(11):2889. doi: 10.3390/biomedicines11112889 (PMC10668975; doi:10.3390/biomedicines11112889)
Supplement: Supplementary file 1 [file biomedicines-11-02889-s001.zip › biomedicines-2678323-supplementary.pdf]

Table S1. Clinical features of CRC patients

Sample ID	Gender	Age at diagnosis	Stage (TNM)	HER-2	CEA	Survival status (Death event=1)	Treatment time (Week)	Recurrence-free survival time (Month)
CRC-01	Female	48	T4N1M0	+	1.12	0	37	63
CRC-02	Male	63	T3N2M0	+	2.4	0	20	46
CRC-03	Female	60	T3N0M0	+	1.14	0	20	6
CRC-04	Female	72	T2N0M0	+	2.02	0	2	60
CRC-05	Male	64	T3N1M0	-	5.07	0	13	61
CRC-06	Male	28	T2N0M0	-	3.02	0	17	61
CRC-07	Female	62	T3N1M0	++	1.89	0	13	72
CRC-08	Male	78	T2N0M0	-	1.48	0	3	62
CRC-09	Male	53	T4N1M0	+	0.92	0	26	60
CRC-10	Male	61	T2N0M0	+	2.1	0	20	60
CRC-11	Female	68	T3N0M0	-	1.05	0	17	60
CRC-12	Male	75	T2N0M0	+	3.01	0	2	12
CRC-13	Male	56	T3N2M0	+	11.16	0	38	41
CRC-14	Female	51	T3N0M0	+	1	0	25	10
CRC-15	Female	50	T4N0M0	+	2.09	0	41	60
CRC-16	Male	48	T1N0M0	-	3.08	0	2	1

CRC-17	Male	40	T3N0M0	-	1.67	0	59	44
CRC-18	Female	69	T1N0M0	-	1.32	0	2	63
CRC-19	Male	61	T2N0M0	-	3.91	0	19	63
CRC-20	Male	68	T4N0M0	-	7.52	0	20	63
CRC-21	Male	63	T2N1M0	-	1.63	0	25	67
CRC-22	Male	45	T3N0M0	-	2.21	0	17	61
CRC-23	Female	57	T3N0M0	++	1.73	0	26	60
CRC-24	Male	62	T3N1M0	-	18.57	0	21	30
CRC-25	Male	58	T4N3M0	+	2.15	0	19	60
CRC-26	Male	73	T4N2M0	+	5.6	0	1	1
CRC-27	Male	59	T3N0M0	+	6.26	0	27	60
CRC-28	Female	71	T4N0M0	-	1.36	0	27	60
CRC-29	Female	62	T4N2M0	-	1.67	0	21	60
CRC-30	Male	54	T4N2M0	-	1.23	0	15	0
CRC-31	Male	73	T2N0M0	-	2.4	0	2	65
CRC-32	Male	70	T4N0M0	+	1.54	0	2	17
CRC-33	Male	57	T4N2M0	+	2.05	1	38	37
CRC-34	Female	45	T3N0M0	+	7.39	0	17	60
CRC-35	Female	56	T2N0M0	+	91.33	0	32	9
CRC-36	Female	50	T3N2M0	-	1.52	0	38	45
CRC-37	Female	67	T3N0M0	+++	1.52	0	26	8
CRC-38	Male	51	T2N1M0	-	1	0	13	9

CRC-39	Female	65	T3N0M0	+	5.32	0	9	60
CRC-40	Male	68	T3N0M0	+	3.11	0	20	64
CRC-41	Female	71	T3N1M0	+	3.54	0	2	0
CRC-42	Female	39	T4N1M0	-	7.32	0	14	60
CRC-43	Female	70	T2N0M0	-	0.9	0	2	1
CRC-44	Female	41	T4N0M0	-	2.22	0	70	18
CRC-45	Female	67	T3N0M0	-	1.29	0	11	26
CRC-46	Male	53	T3N0M0	+	1.66	0	38	63
CRC-47	Male	68	T2N0M0	+	23.97	0	10	3
CRC-48	Male	51	T3N0M0	-	2.05	0	26	12
CRC-49	Male	47	T4N2M0	++	1.01	0	16	12
CRC-50	Male	69	T3N0M0	+	125	0	21	60
CRC-51	Female	63	T2N0M0	-	7.46	0	19	60
CRC-52	Male	61	T3N1M0	+	0.93	0	73	17
CRC-53	Male	53	T2N0M0	-	6.94	1	17	56
CRC-54	Male	43	T2N1M0	+	0.89	0	14	63
CRC-55	Female	61	T3N2M0	-	3.82	0	35	63
CRC-56	Male	48	T2N0M0	+++	13.23	0	23	64
CRC-57	Male	66	T4N1M0	-	3.77	0	26	61
CRC-58	Female	46	T2N2M0	-	0.66	0	21	8
CRC-59	Male	56	T3N0M0	+	36.65	1	34	60
CRC-60	Male	85	T4N1M0	-	6.77	1	2	53

CRC-61	Male	62	T4N0M0	-	3.35	1	38	39
CRC-62	Male	59	T3N1M0	-	5.62	0	26	62
CRC-63	Male	62	T3N0M0	-	5.24	0	28	12
CRC-64	Male	59	T4N1M0	-	3.56	0	10	10
CRC-65	Male	47	T3N1M0	++	10.5	0	10	40
CRC-66	Female	79	T3N1M0	+	9.41	0	26	60
CRC-67	Male	39	T3N0M0	+	12.23	0	16	8
CRC-68	Male	47	T3N2M0	-	4.43	0	17	60
CRC-69	Male	53	T4N1M0	+	93.93	1	19	65
CRC-70	Female	64	T2N0M0	-	16.57	1	21	53
CRC-71	Male	62	T2N0M0	+	7.6	1	16	61
CRC-72	Male	57	T3N0M0	-	9.74	1	25	46
CRC-73	Female	44	T3N1M0	-	2.51	0	20	61
CRC-74	Female	59	T3N2M0	-	0.71	0	32	60
CRC-75	Female	73	T3N0M0	-	8.87	0	2	61
CRC-76	Male	58	T3N2M0	-	16.18	0	32	40
CRC-77	Female	35	T4N2M0	-	63.35	0	26	12
CRC-78	Female	74	T4N1M0	++	5.05	0	20	12
CRC-79	Female	63	T4N0M0	-	1.03	0	17	42
CRC-80	Male	51	T2N0M0	+	0.84	0	26	12
CRC-81	Male	71	T3N0M0	-	1.85	0	2	60
CRC-82	Female	50	T3N1M0	-	5	0	20	7

CRC-83	Male	81	T4N1M0	+	8.73	0	2	60
CRC-84	Male	77	T4N1M0	-	44.92	1	2	27
CRC-85	Male	50	T4N0M0	+	1.08	0	38	60
CRC-86	Male	53	T4N2M0	+	3.43	0	28	7
CRC-87	Female	47	T2N2M0	-	2.33	0	55	21
CRC-88	Female	61	T3N0M0	++	94.79	0	21	61
CRC-89	Female	63	T3N1M0	+	3.7	0	20	7
CRC-90	Male	56	T3N2M0	-	5.67	0	38	7
CRC-91	Male	81	T4N1M0	+++	4.06	0	27	15
CRC-92	Female	36	T4N2M0	+	5.32	0	26	29
CRC-93	Female	54	T4N0M0	++	1	0	38	62
CRC-94	Female	66	T3N1M0	+	1.35	0	20	24
CRC-95	Male	63	T3N1M0	+	1.31	0	25	10
CRC-96	Male	61	T4N0M0	-	51.33	1	17	8
CRC-97	Female	41	T4N0M1	-	392.7	0	70	18

*: The degrees of differentiation were defined from low to high: 1: low differentiation; 2: medium-low differentiation; 3: medium differentiation; 4: medium-high differentiation; 5: high differentiation

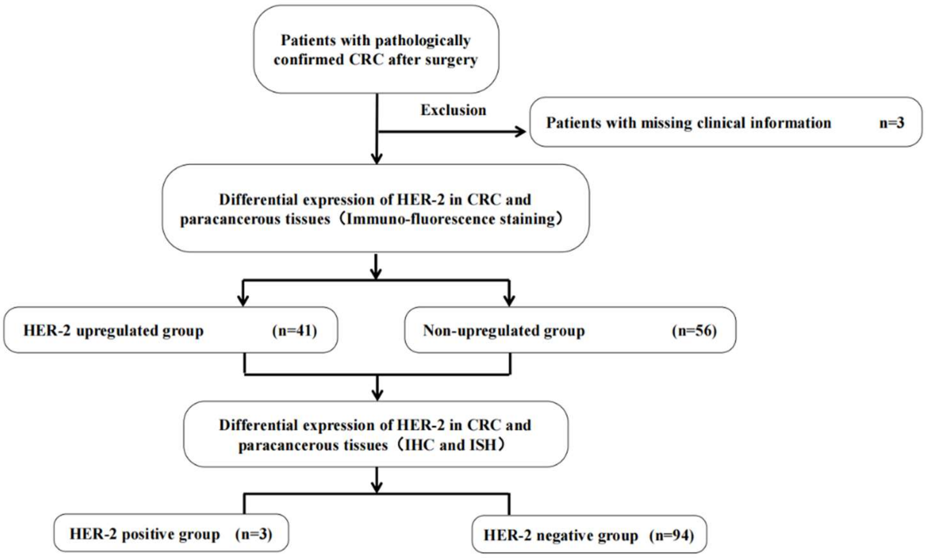

Fig. S1 Patient intake and grouping flowchart

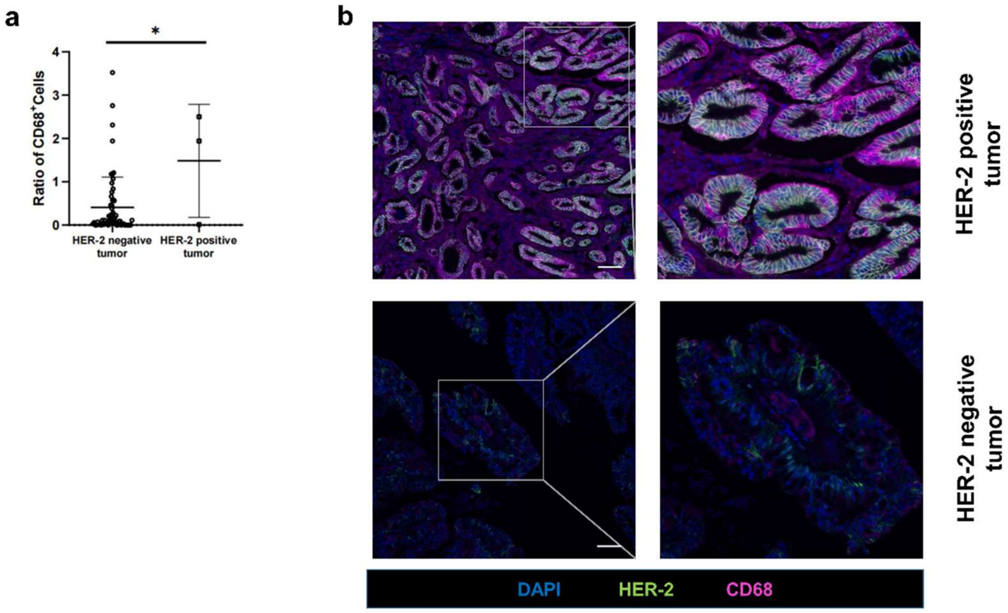

Fig. S2 Expression of CD68 in HER-2-positive and HER-2-negative CRC tissues. Fig. S2a: Increased expression of CD68 in HER-2 positive CRC tissue. Fig. S2b: CD68 immunofluorescence in HER-2 positive and negative tissue. Scale bar: 100 μm. *: $p<0.05$.
